# Fracture of the Skull, with Laceration of Membranes and Injury to Brain

**Published:** 1872-09

**Authors:** B. F. Chambliss

**Affiliations:** Russellville, Georgia


### FRACTURE OF THE SKULL, WITH LACERATION OF MEMBRANES AND INJURY TO BRAIN.

BY B. F. CHAMBLISS, M.D., RUSSELLVILLE, GEORGIA.

[Read Before the Middle Georgia Medical Society, July 17th, 1872.]

June 7th, 1872, I was summoned to see Hardy, a negro man aged twenty-five years, of robust constitution—previous health good, but naturally of weak mind—who was reported to have been struck by another freedman with a weeding hoe. On my arrival, I found him lying on the floor of a cabin, where he was placed when brought from the field. On inquiry, I learned that he was insensible for half an hour or more after receiving the blow. I was also informed by Mrs. S., an intelligent lady, that he had had several convulsions since he was brought to the house.

Upon examination, I found that he was suffering very much; could articulate very indistinctly when questioned, and had rather a slow pulse. After further examination, I found the scalp was wounded about three inches in length, across the frontal bone of the left side, near its junction with the temporal, and about an inch from, and running almost parallel with, the coronal suture. I also discovered that the frontal bone had been broken through for ten and a half inches in length,

The edges of the wound through the soft integument were smooth, though torn loose on either side for half an inch, and from the appearance, it must have been done with the round part of the blade, near the eye of the hoe, and as there was no depression of the skull, I do not think the eye struck the head at all. The fracture through the bone was large enough for me to insert my little finger of my right hand to the second joint, when I found some loose spiculæ of bone just within the wound, which were very easily removed. But upon a closer examination, I discovered there were some fragments within the cranium, all of which were readily taken out, except one piece, which was partially imbedded in the brain. This fragment I succeeded in removing, after some little time and difficulty, by elevating one corner with my little finger sufficiently to get hold of it with a pair of arterial forceps. This piece was the largest that was found—about three-fourths of an inch long, and half an inch wide. It consisted of a part of the inner tablet, which was partly scaled off from the outer table, which still remains intact.

I found the membranes were considerably lacerated, and a portion of the cerebral substance escaped before and after the removal of the fragments of bone. After removing all the foreign bodies I could find, I took off the hair for some distance around the wound, which I left open for the escape of any foreign substance that might still remain, or should form during the progress of treatment. I directed him to lie on his left side, so as to facilitate the discharge of anything that might be injurious to the brain. I also ordered cold-water dressings to be constantly applied, which I prefer to all other applications (with my limited experience) in wounds of this nature. I will here remark, that his right side was completely paralyzed—his tongue partially so; and after the removal of the spiculæ of bone, he seemed to experience considerable relief, from which time he had no more convulsions.

On the morning of the 8th, I saw him again; learned that he had rested tolerably well the night before, and was then quiet, with no excitement of the circulation. Continued the same treatment, with directions for him to have a dose of epsom salts in the afternoon, and to be repeated whenever necessary.

I visited him again on the 10th; found him comfortable—no excitement about the circulation—but was informed by Dr. S., of Alabama, who was on a visit to his brother, that there was considerable excitement on the night of the 8th, but it passed off the following morning.

I again saw him on the 13th, and found him rather stupid; pulse slow; tongue paralyzed—could move it but little, and could scarcely be understood when speaking. He seemed to be suffering from pressure of the brain. I inserted my finger in the wound carefully, and separated the edges, when a small quantity of pus escaped, from which he seemed to obtain some relief.

I also saw him on the 14th, 16th and 19th, at which times he seemed to be doing very well. On the 19th, I discovered he could move his right leg a little, but not sufficient to walk without assistance.

Hearing from him occasionally, and learning that he was getting on pretty well, I did not see him again until the 6th of July, when I was informed that he had been walking about a good deal too much, as he was compelled to take his bed again for a short time.

I visited him again on the 11th, when I found that he was able to walk about, though his step was still unsteady. During this visit, I discovered there was a soft substance within the wound, which I at first took to be a fungus growth from the brain, but found I was mistaken, after a careful examination. It seemed to be a coalescence of exuberant granulations. After judging this to be the case, I cauterized freely with the solid nitrate of silver, and directed it to be applied every morning.

At my next visit, which was on the 15th, I found the growth but little diminished. I concluded it would be best to pare off the growth before applying the caustic, which I did two or three times. After this, it diminished more rapidly.

On the 1st of August, I discovered there was a loose spiculæ of bone just within the wound, which had nearly closed up, which piece was easily removed, and the only fragment that has been found since the first dressing. Since the removal of this piece, the wound has entirely healed.

He has not complained of any pain of the head for some time, and his mind is as good as it was prior to the infliction of the wound. His general health now (August 20th) is very good, and he walks several miles in a day, though he can not use his right leg as well as he did before being wounded. He has but little use as yet of his right arm and hand; he can raise his hand as high as his shoulder, but has no use of his fingers.

I will state, in conclusion, that there was considerable hemorrhage when I first saw him, which soon ceased after the fragments had been removed and cold water applied. I enjoined low diet throughout the treatment, which was strictly observed until he began to walk about. When I first saw the patient, I had but little hope of his recovery, for it was very evident that the brain and its membranes had sustained considerable injury. Now, the question naturally arises—Will he ever recover the use of his right arm entirely? The answer to which I will leave to others and the future.

